# Explorative Insights into Local Immune Response to BK Virus—A Cross-Sectional Study in Urine Samples Between Transplant Recipients and Non-Immunocompromised Hosts

**DOI:** 10.3390/medicina62020240

**Published:** 2026-01-23

**Authors:** Agata Michnowska, Bartosz Wojciuk, Paulina Reus, Agata Filipowska, Magdalena Mnichowska-Polanowska, Bartłomiej Grygorcewicz, Kazimierz Ciechanowski, Karolina Kędzierska-Kapuza

**Affiliations:** 1 Department of Immunological Diagnostics, Pomeranian Medical University, 70-204 Szczecin, Poland; 2 Clinical Department of Internal Diseases, Nephrology and Transplantology, Pomeranian Medical University, 70-204 Szczecin, Poland; 3 Department of Information Systems, Institute of Informatics and Quantitative Economics, Poznań University of Economics and Business, 61-875 Poznan, Poland; 4 Department of Clinical Microbiology, Pomeranian Medical University, 70-204 Szczecin, Poland; 5 Department of Genomics and Forensic Genetics, Pomeranian Medical University, 70-204 Szczecin, Poland; 6 Centre of Diabetology, National Medical Institute of the Ministry of Interior Affairs and Administration, 02-507 Warsaw, Poland

**Keywords:** BK Polyomavirus, kidney transplantation, urine, proteomics, immune response, KLRD1 protein, CD94 antigens, cross-sectional studies, DNA Viruria, urinary proteomics

## Abstract

*Background and Objectives*: BK virus (BKPyV) is a common latent pathogen in humans, but it becomes particularly insidious in kidney transplant recipients, where reactivation may contribute to allograft loss. The immune mechanisms controlling BKPyV latency in immunocompromised hosts remain incompletely understood. We assume the urinary immune proteome reflects local immune response in the kidney and the urinary tract. Thus, this study aimed to determine whether the presence of BKPyV alters the urinary immune-related proteomic profile of kidney transplant recipients and shifts it away to that observed in healthy individuals. *Materials and Methods*: 137 urine samples were collected from kidney recipients, both BKPyV-positive and BKPyV-negative, patients with stage 5 chronic kidney disease, and healthy controls. Targeted proteomic analysis was performed using the proximity extension assay, followed by heatmapping, principal component analysis, random forest, and linear regression modeling. *Results*: The urinary proteome of BKPyV-positive recipients remained more distinct from healthy controls than that of BKPyV-negative ones. Among the 33 proteins detected across all samples, 17 showed significant intergroup differences, with KLRD1 (CD94) uniquely upregulated in all transplant recipients, but downregulated in BKPyV-positive samples. *Conclusions*: We conclude that the presence of BKPyV in the urinary tract of kidney recipients notably interplays with the local immune response even in the absence of clinical disease.

## 1. Introduction

The BK virus (BKPyV) represents members of the *Polyomaviridae* family. First isolated in 1971, this is a small, non-enveloped DNA virus with a circular, double-stranded genome. BKPyV infection is widespread in the general population, with seroprevalence rates ranging from 60% to 80% in adults globally [1]. After primary exposure, BKPyV primarily establishes a latent infection in the renal epithelium, and this is generally asymptomatic in immunocompetent individuals. It is well-documented that transient DNAuria, characterized by the shedding of BKPyV in the urine, can occur sporadically even in healthy individuals without any clinical consequences [2]. Nevertheless, in kidney recipients, BKPyV reactivation occurs mainly during the first two years after kidney transplantation with potentially severe complications, including hemorrhagic cystitis, ureteric stenosis, and most notably, BK virus-associated nephropathy (BKVAN) [3]. The risk of such complications ranges from 1 to 10% [4]. However, the replication of the BK virus itself does not always result in the onset of full-blown BKVAN. Asymptomatic DNAuria followed by DNAemia represents the preceding stages. Asymptomatic BKPyV DNAuria affects 20–60% of kidney recipients, with 13% of these individuals subsequently developing BKPyV DNAemia. Detection of BKPyV viral load in the blood in combination with impaired graft function constitutes a clinical suspicion of BKVAN.

The laboratory diagnostics of BKPyV infection and associated complications rely on various techniques, including viral DNA detection in body fluids using polymerase chain reaction (PCR) as mentioned earlier, and histopathological examination of renal biopsies supplemented with SV40 large T antigen staining [5]. The updated 2024 guidelines for BKPyV infection management in kidney recipients recommend routine, monthly plasma surveillance with quantitative BKPyV DNA PCR during the first nine months post-transplant, followed by every three months testing up to 24 months post-transplant, and annually or as clinically indicated, thereafter. A plasma viral load exceeding 1000 copies per milliliter is considered indicative of the need for closer monitoring, while persistent or rising levels above 10,000 copies per milliliter warrant further diagnostic evaluation. In cases of significant DNAemia or unexplained graft dysfunction, the renal biopsy remains the definitive diagnostic tool, with histological evidence of viral cytopathic changes and positive SV40 large T antigen staining used to confirm BKVAN. Although urine cytology and urinary PCR may support early detection of reactivating BKPyV, plasma PCR is regarded as the gold standard for guiding the clinical management [2].

BKPyV DNAuria appears transiently in both kidney recipients and in healthy individuals. However, it seems to be more frequent and may potentially lead to clinical consequences only under immunosuppression. Despite the advances in transplant medicine and antiviral therapies, the management of BKPyV-associated complications remains challenging, with limited therapeutic options and variable outcomes. Thus, there is a growing need for an improved understanding of how BKPyV reactivation is initiated. Although the immunological mechanisms underlying polyomavirus infection remain incompletely understood, recent research has begun to shed light on the immune pathways involved in viral control and tissue-specific immune activation. Experimental and translational studies suggest that BKPyV reactivation may trigger distinct antiviral immune responses within the kidney microenvironment, including chemokine-mediated recruitment of T cells and the modulation of cytotoxic and regulatory cell subsets. Emerging evidence also suggested potential interactions between signaling axes such as CXCR6–CXCL16, and other immune receptors, including CD94, which may reflect the development of specialized T cell phenotypes within tissues affected by BKPyV replication [6]. Hence, we hypothesized that detectable BKPyV DNAuria associates with the profile of immune response markers present in the urine of kidney recipients and that this is possibly dependent on the urine viral DNA load.

Proteomic research explores the entire set of proteins expressed in a cell, tissue, or organism at a specific time point. In recent years, advanced molecular approaches, including next-generation sequencing (NGS) and other omics-based techniques, have enabled comprehensive characterization of viral genomes, viral diversity, and host–virus interactions [7]. However, nucleic acid-based methods primarily reflect the presence and quantity of viral genetic material, whereas proteomics provides complementary insight into downstream functional immune responses at the protein level [8]. Urine proteomics appears particularly promising due to its non-invasive character and, at the same time, due to its explorative potential in reflecting local changes in the urinary tract. At the same time, the Olink^®^ Target 96 Immuno-Response panel is a multiplex platform that enables simultaneous quantification of 92 targeted immune-related proteins involved in a range of local immune response processes, such as inflammation, cell-mediated cytotoxicity, immune cell activation, and antiviral defense.

The primary aim of the study was (1) to explore the differences between the profiles of immune mediators in the urine of kidney transplant recipients with and without BKPyV-DNAuria; (2) to determine whether BKPyV post-transplant shifts these profiles away from healthy individuals. Secondarily, we aimed to determine whether the differentially expressed proteins detected in the urine in kidney recipient’s groups reflect biological relevance in controlling BKPyV latency.

## 2. Materials and Methods

### 2.1. Subject

This study was designed as an exploratory cross-sectional comparative study. The study aimed to characterize differences in local urinary immune proteomic profiles across these groups without longitudinal follow-up or interventional procedures. The study was conducted and reported in accordance with the STROBE guidelines for cross-sectional studies.

The study was conducted at the Clinical Department of Internal Medicine, Nephrology and Transplantation, in collaboration with the Department of Immunological Diagnostics of Pomeranian Medical University in Szczecin and the National Medical Institute of the Ministry of the Interior Affairs and Administration in Warsaw. The study was approved by the Local Bioethical Committee (decision number KB-0012/136/17) and written consent was obtained from all participants. Urine samples were collected at a single time point from adult participants belonging to predefined study groups, between 6 and 12 months after kidney transplantation. We collected 104 samples, from 51 kidney transplant recipients, 18 samples from 18 CKD patients and 15 samples from 15 healthy controls, between 2018 and 2022. The sample size was determined by the availability of urine samples that met the predefined eligibility criteria.

**Scheme 1:**
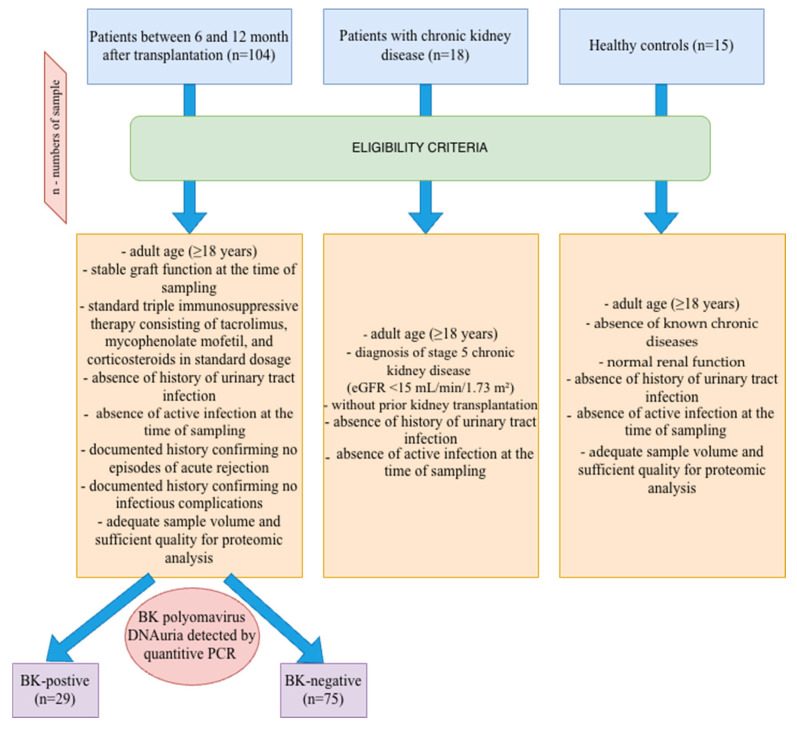
Flow chart illustrating study group allocation and sample selection process. It also presents the number of urine samples included at each step of the study. The corresponding number of patients is provided in Table 1.

All participants were adults (≥18 years of age) representing immunocompromised and non-immunocompromised hosts. Kidney transplant recipients with BKPyV DNAuria (TX-BK+/BK+; group 1) and kidney transplant recipients without BKPyV DNAuria (TX-BK−/BK−; group 2) both represented immunocompromised hosts; whereas patients with stage 5 chronic kidney disease (CKD; group 3) and healthy controls (HC; group 4) represented non-immunocompromised hosts. The flowchart indicating groups selection is presented in Scheme 1 and the detailed baseline characteristics of the study population are presented in Table 1.

Since the Olink^®^ Target 96 Immuno-Response panel has not previously been applied to urine samples, four study groups, as aforementioned, were designed to provide a comprehensive comparison between urinary proteomic profiles. Patients with stage 5 chronic kidney disease were indicated to the whole group of kidney transplant recipients (combined BK+ and BK−) to identify the proteomic shifts related to kidney transplantation itself. This stage preceded and created the background for the targeted comparison between both groups of kidney transplant recipients (BK+ and BK−).

### 2.2. Urine Preparation

Samples of fresh voided urine from the patients were centrifuged for 20 min at 2000 rpm directly after collection. The supernatant was then stored at −80 °C until further analysis. All samples were verified regarding BKPyV DNAuria onset using GeneProof BK Virus PCR Kit (GeneProof a.s., Brno, Czech Republic). Preceding DNA isolation was performed using the standardized nucleic acids extraction system from Sacace Biotechnologies—SaMag12 (Sacace Biotechnologies S.r.l., Como, Italy), according to the manufacturer’s instructions.

### 2.3. Protein Profiling

The samples were thawed at 4 °C, shipped, and processed in Olink^®^ Proteomics (Uppsala, Sweden), using the Immune Response panel. In total, 100% of the samples passed the quality control. The results were expressed in Normalized Protein Expression (NPX) units, which is a normalized, log-transformed measure of protein levels in samples.

### 2.4. Computational Analysis

All analyses were performed using RStudio (version 1.3.1093) with R (version 4.0.3) and Python (version 3.11). The overall computational strategy combined exploratory data visualization, unsupervised clustering, supervised classification, dimensionality reduction, and classical regression modeling. These analytical approaches are complementary, providing both global and targeted perspectives on proteomic variation across study groups.

#### 2.4.1. Protein Expression Mapping

To explore global patterns of urinary immune protein expression across the study groups—healthy controls, chronic kidney disease, kidney transplant recipients with and without BKPyV—heatmaps were generated using the “ComplexHeatmap” package (version 2.6.2) in R. NPX values were standardized by z-score transformation. Hierarchical clustering based on Euclidean distance was applied to display patterns of differentially expressed proteins (DEPs) among samples after z-score transformation of NPX values. DEPs were split using k-means clustering.

#### 2.4.2. Venn Diagram

A Venn diagram was used to illustrate the differences between groups and was performed using the open-access tool available on https://bioinformatics.psg.ugent.be/cgi-bin/liste/Venn/calculate_venn.html (accessed on 21 February 2024).

#### 2.4.3. Random Forest Classification and Feature Selection, Principal Component Analysis

Prior to downstream machine learning analysis, the protein expression matrix was standardized using StandardScaler, as the protein variables had diverse units and measurement scales. The K-Means algorithm was then executed iteratively for a range of cluster numbers *k* ∈ [2, 10]. The optimal number of clusters was selected based on the value of k that maximized the silhouette score. The final K-Means model generated cluster labels for each sample based on this optimal configuration.

Subsequently, a Random Forest (RF) classifier was trained on the same scaled protein dataset, using the cluster labels from K-Means as the target variable. Random Forest—an ensemble method based on aggregating of multiple decision trees—is well-suited for capturing complex and nonlinear interactions between variables [9]. After training, feature importance scores were computed. These scores reflect the relative contribution of each variable to the tree-based decision-making process. High feature importance values indicate that the corresponding protein was a significant contributor to defining the clustering structure.

To visualize overall similarities and differences in protein expression across groups, with dimensionality reduction, Principal Component Analysis (PCA) was performed using the subset of proteins identified as important by RF.

#### 2.4.4. General Linear Modeling and Statistical Testing

**Scheme 2:**
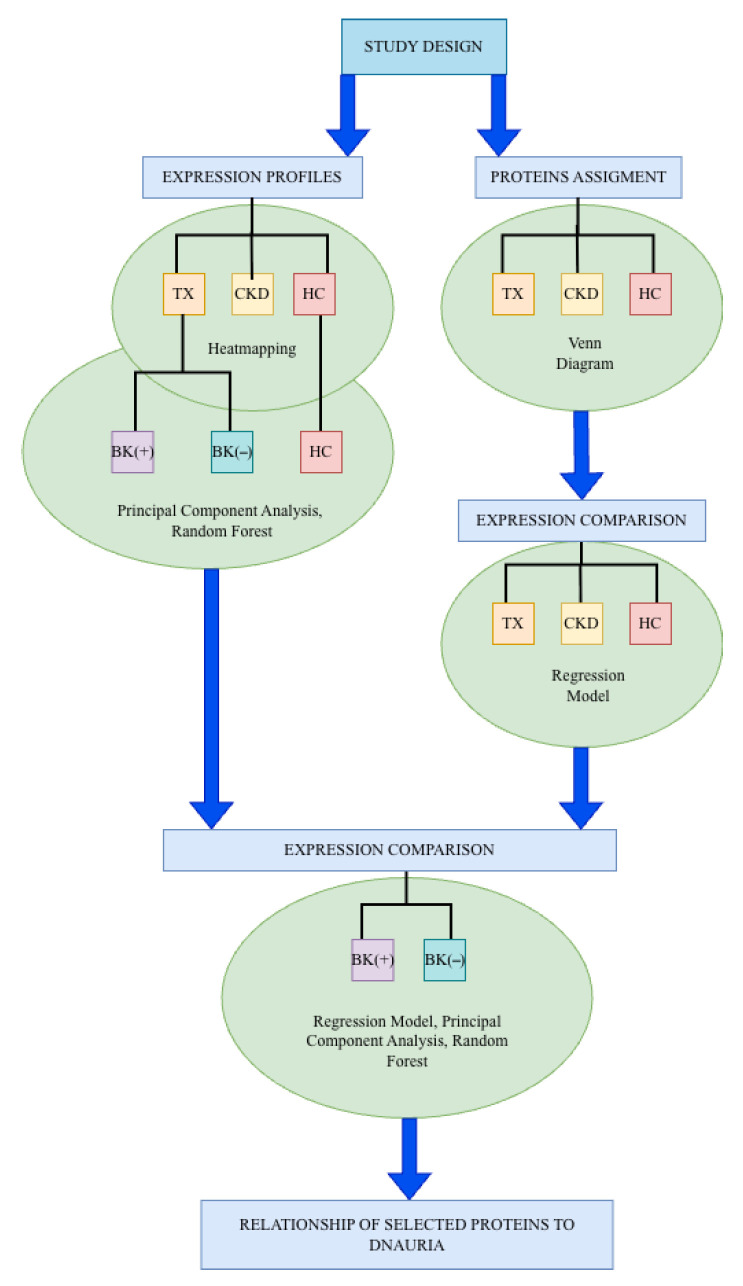
Flow chart illustrating analytical pipeline.

Classical statistical modeling was conducted to confirm differential protein expression. Calculated NPX values above the respective protein limit of detection (LOD) in at least 30% of samples were included in further analysis. A general linear model (GLM) regression approach with analysis of contrasts, with FDR correction by Benjamini–Hochberg, using the R package “emmeans” (version 1.6.2.1) was performed. GLM was fitted to the expression of each protein. As previously mentioned, we performed two approaches. First, we targeted the following sample groups: TX (including both BK-positive and BK-negative), HC, and CKD; GLM was fitted using the condition/group as the independent variable and sex as a confounding factor. Then, we targeted the TX group, which was divided into BK(+) and BK(−) groups. GLM was fitted using the presence/absence of BK infection within the TX group as an independent variable considered differentially expressed with a *p*-value < 0.05. Ggplot2 (version 3.3.5) was used for graphics generation. The differentially expressed proteins representing the detection rates over 90% were included into further investigation which targeted the relationship between NPX and DNAuria load. Pearson correlation rate was used in these calculations. DNAuria values were log transformed. The statistical framework was based on methodologies described in previous studies, analogously approaching Olink^®^ protein panels [10]. The analytical pipeline implemented in this study is presented in Scheme 2.

## 3. Results

### 3.1. BKPyV Detection

**Figure A1:**
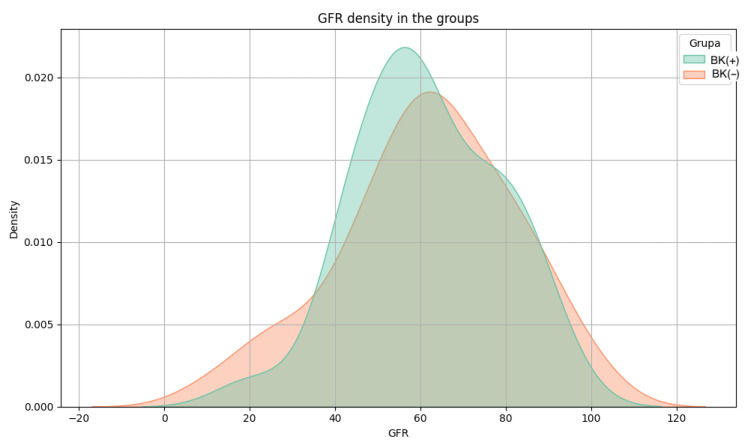
Density distribution of GFR in the groups. Density plots show the distribution of estimated GFR values among kidney transplant recipients without (BK−) and with (BK+) detectable BKPyV DNAuria.

BKPyV DNAuria was detected in 29 out of 104 samples (27.9%). Based on this, we divided the samples into respective groups: BK(+) and BK(−). The differences in glomerular filtration rate (GFR) values between the BK(−) group and the BK(+) group were not statistically significant at the moment of sample collection (Table 2, Figure A1). The values of BKPyV DNA load are presented in Table A1.

### 3.2. Proteins Detectability

Out of the 92 proteins targeted within the immune response panel, 82 were detected in the analysis, 24 showed a detection rate above 30% of all tested urine samples, 36 exceeded 50% in at least one of the groups, and 16 had over 90% detectability—including 11 that were detected in 100% of samples in at least one group. A complete list of proteins assigned to groups with detection rates is presented in Table S1 of the Supplementary Materials.

### 3.3. Protein Expression Profiles

**Figure A2:**
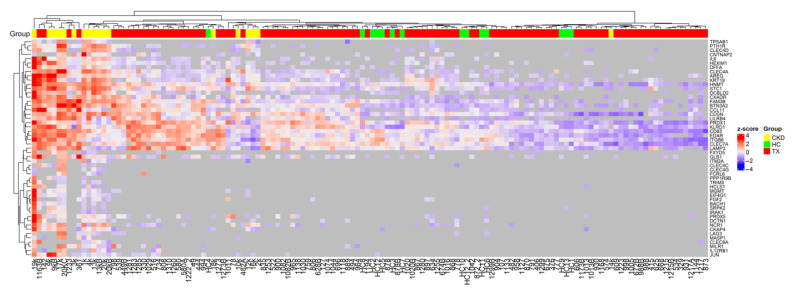
Hierarchical clustering heatmap of proteins across study groups. The heatmap visualizes standardized expression levels (Z-scores) of proteins in urine samples from patients with chronic kidney disease (CKD), kidney transplant recipients (TX), and healthy controls (HC). Rows represent proteins and columns represent individual samples.

The hierarchical clustering heatmap illustrates the standardized expression levels across all samples from the following experimental groups: TX (combined BK+ and BK−), CKD and HC (Appendix, Figure A2). The numbers of subclusters have been extinguished, with the TX group distributed among all of these.

**Figure 1:**
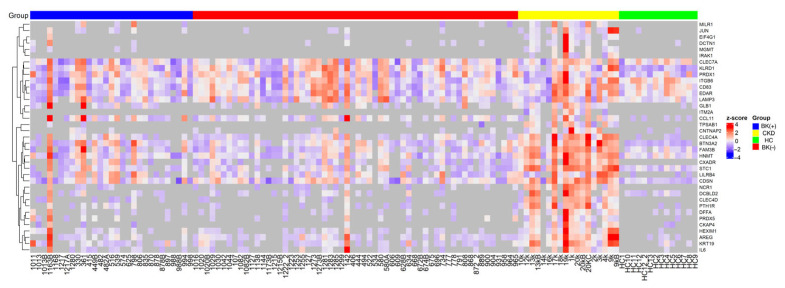
Heatmap for BK(+), CKD, HC, and BK(−) groups. The z-score normalized NPX values are color-coded from red (high expression) to blue (low expression). Gray cells represent proteins not detected in each sample. Columns represent individual samples; rows represent proteins. Groups are indicated by colored bars above the heatmap: blue—BK-positive kidney transplant recipients (BK+), red—BK-negative transplant recipients (BK−), green—healthy controls (HC), and yellow—patients with chronic kidney disease (CKD).

We further used a heatmap to demonstrate the protein expression profiles in the respective targeted groups of CKD, HC, BK(−), and BK(+) (Figure 1). The overall distribution of immune-related urinary proteins revealed distinct expression patterns among the analyzed groups. Healthy controls demonstrated consistently low protein abundance, while CKD samples showed high protein abundance. Kidney transplant recipients—both BK+ and BK—remained internally differentiated.

Since the heatmaps were used to illustrate up- and downregulation of all detected proteins irrespectively of their detection rate, the statistical significance was not considered. To gain a deeper insight beyond what could be observed in the heatmap, we applied a dimensionality reduction strategy to explore the underlying structure of the proteomic data.

**Figure 2:**
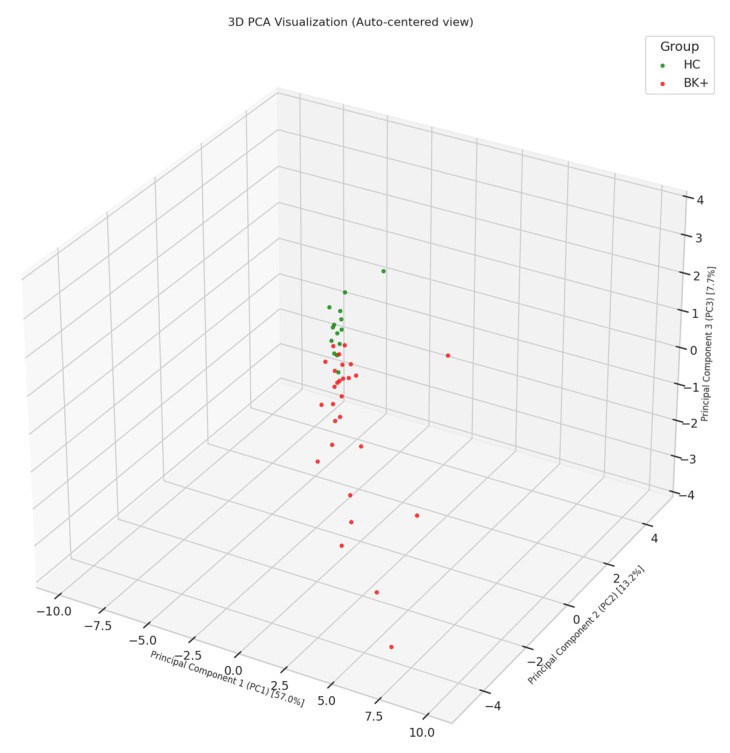
PCA visualization with group-specific areas. The plots illustrate how individual samples are distributed based on principal component analysis of the most informative urinary proteins. BK(+) kidney transplant recipients compared to healthy controls. BK(+) recipients displayed greater variability and partial separation, suggesting that virus-related modulation of immune protein expression occurred. The first three principal components explained 77.9% of the total variance (PC1: 57.0%, PC2: 13.2%, PC3: 7.7%).

**Figure 3:**
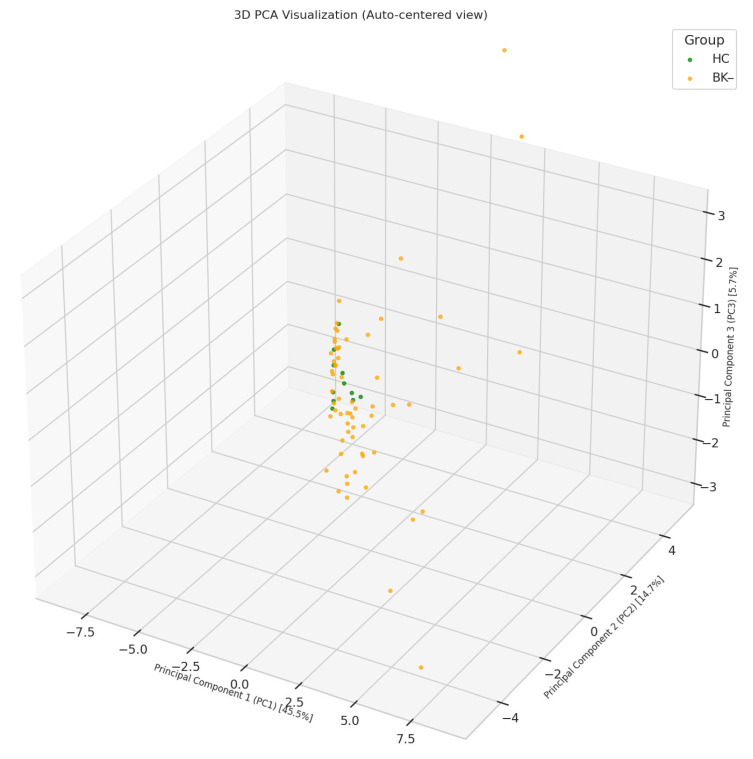
PCA visualization with group-specific areas. The plots illustrate how individual samples are distributed based on principal component analysis of the most informative urinary proteins. BK(−) recipients showed profiles that more closely resembled those of HC. The explained total variance was 69.9% (PC1: 45.5%, PC2: 14.7%, PC3: 5.7%).

Three-dimensional PCA visualization revealed distinct clustering patterns among the analyzed groups. Figure 2—the BK(+) kidney transplant recipients and healthy controls showed clear separation, with a minimal overlap, with most HC samples forming a compact cluster. Figure 3—the BK(−) kidney transplant recipients exhibited the protein expression pattern overlapping substantially with HC indicating closer similarity to their proteomic profile. This analysis was performed using the selected proteins with a detection rate of at least 50% in at least one of the study groups

Random Forest analysis was employed to identify the most informative proteins contributing to group discrimination based on their importance scores. The resulting rankings revealed distinct sets of proteins differentiating BK(+) and BK(−) kidney transplant recipients from HC and indicating that the discriminative proteomic signatures were not identical between the two transplant groups. In the BK(+) vs. HC comparison revealed that KLRD1 (CD94) emerged as one of the top-ranked features. The complete results of the Random Forest analysis are provided in Supplementary Materials Figures S1 and S2.

### 3.4. Proteins Assignment

**Figure 4:**
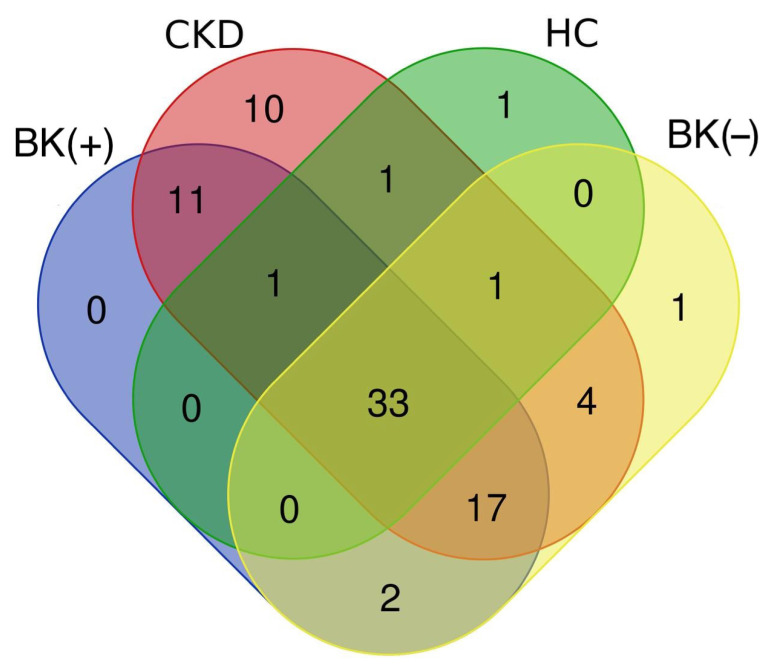
Venn diagram. Venn diagram illustrating the overlap of detected urinary proteins among four study groups: BKPyV-positive transplant recipients (BK+), BKPyV-negative transplant recipients (BK−), chronic kidney disease patients (CKD), and healthy controls (HC). Each region of the diagram indicates the number of proteins uniquely or commonly shared between the specified groups. The largest overlap (33 proteins) was observed among all four clinical groups.

We identified a certain number of proteins that appeared either characteristic of each group or were commonly shared between different groups. The detailed results are presented in a Venn diagram (Figure 4). The largest number of shared elements—33, was presented as common across all groups, including 16 with a detection rate above 90% (CLEC7A, KLRD1, CD83, ITGB6, PRDX1, HNMT, CDSN, EDAR, BTN3A2, LAMP3, STC1, FAM3B, KRT19, CLEC4A, AREG, PTH1R); 17 were shared exclusively among the CKD, BK(+), and BK(−) groups, among these CXADR appeared absent in HC and showed the highest detection rate within this subset, observed in at least 55% of samples. These and the 33 common proteins are listed in Table A2. Eleven proteins were presented as shared between BK(+) and CKD; however, they were detected in single BKPyV-DNA positive samples.

**Figure 5:**
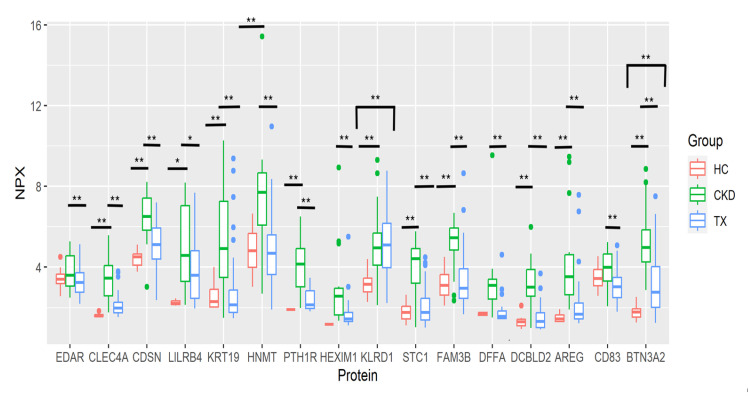
Box and whisker plots with normalized protein expression of significant proteins between HC, CKD, and TX. Boxplot showing the distribution of selected urinary protein in three groups: healthy controls (HC, red), patients with chronic kidney disease (CKD, green), and kidney transplant recipients (TX, blue). * and ** indicate statistically significant with an adjusted *p*-value < 0.05 and <0.01, respectively.

In the regression model, 17 of the 33 common elements were identified as differently expressed between HC, TX, and CKD (Figure 5, the corresponding *p*-values for all comparisons are provided in the Supplementary Materials Tables S2–S4). All the proteins were found downregulated in the healthy controls, with most exhibiting low expression levels. Sixteen of these were upregulated in patients with chronic transplant disease, and one, KLRD1 (CD94), in the kidney transplant recipients. The upregulation of KLRD1 between TX and HC groups was highly significant, and present, but insignificant, between TX and CKD. EDAR, CLEC4A, CDSN, LILRB4, KRT19, HNMT, PTH1R, STC1, FAM3B, DFFA, DCBLD2, AREG, CD83, and BTN3A2 appeared significantly downregulated in kidney transplant recipients compared with CKD patients. Among the differentially expressed proteins, several—including BTN3A2, AREG, CDSN, and PTH1R—showed a gradual increase in expression across the HC, TX, and CKD groups. Except for PADI2, all listed proteins exhibited a detection rate of ≥50% in at least one study group and these have been included in the relevant boxplot.

### 3.5. Differences Between BK(+) and BK(−) Groups

**Figure 6:**
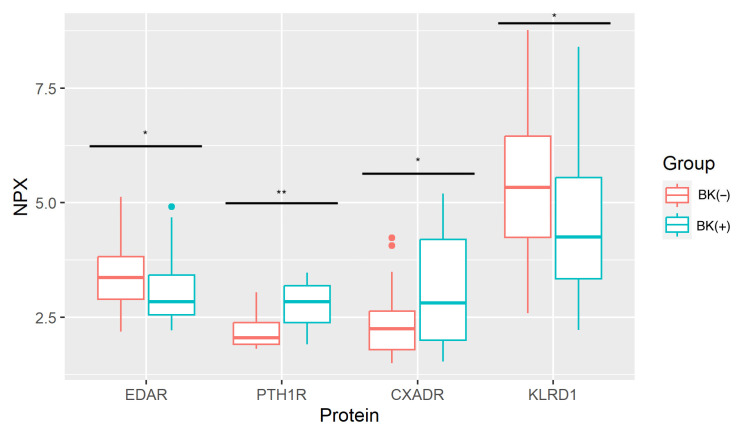
Box and whisker plots with normalized protein expression of statistically significant proteins, BK(−) vs. BK(+). Boxplots show the distribution of urinary protein levels for EDAR, PTH1R, CXADR, and KLRD1 in kidney transplant recipients without BKPyV DNAuria (BK(−), red) and with BKPyV DNAuria (BK(+), blue). * and ** indicate statistically significant with an adjusted *p*-value < 0.05 and <0.01, respectively.

Regression modeling for the BK(−) and BK(+) groups exclusively revealed four proteins with significantly different expression: EDAR, PTH1R, KLRD1, and CXADR. Two of these, EDAR and KLRD1, appeared to be upregulated in the BK(−) group. In contrast, the other two, PTH1R and CXADR, were upregulated in the BK(+) group, respectively (Figure 6). Their pathway assignment has been presented in Table 3.

**Figure 7:**
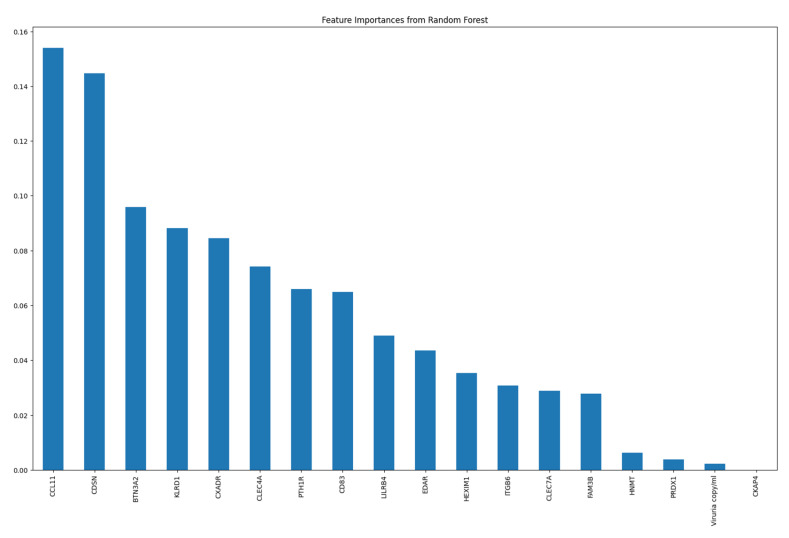
Random Forest BK(+) vs. BK(−). The bar plot illustrates the relative importance of the top urinary proteins that contribute to group separation. CCL11, CDSN, and BTN3A2 were identified as the most informative features, followed by KLRD1 and CXADR. The importance values reflect each variable’s contribution to the model’s accuracy in classifying samples. “Viruria (copy/mL)” represents the input value of BKVyP viral load.

The top-ranking proteins in the Random Forest that distinguish the BK(+) and BK(−) groups include KLRD1, CXADR, and PTH1R, accompanied by CCL11, CDSN, BTN3A2, and CLEC4A. Notably, urinary viral load values used in this model, despite enhancing group separation, did not show high feature importance but rather increased the importance of particular proteins, suggesting their expression as closely associated with BK virus replication (Figure 7).

**Figure 8:**
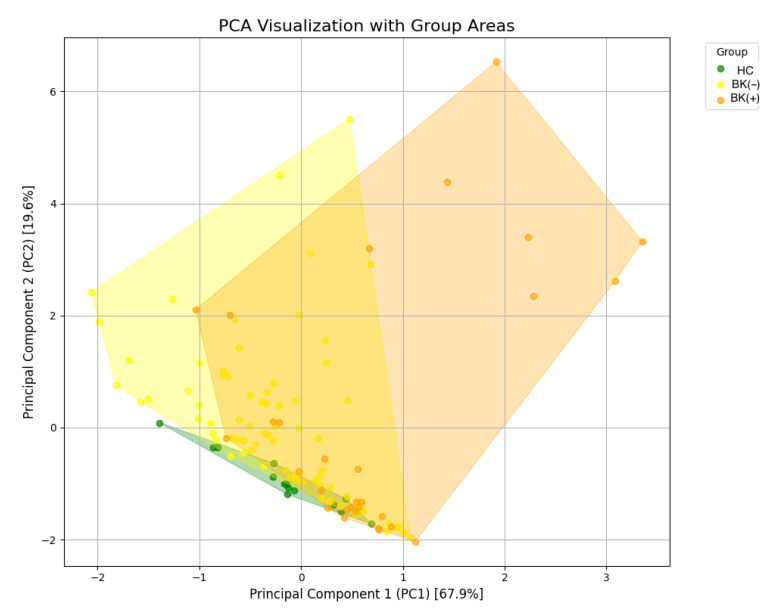
PCA visualization for BK(+) vs. BK(−) vs. HC. Principal component analysis was performed based on the expression of selected urinary proteins to visualize differences between healthy controls (HC, green), kidney transplant recipients without BKPyV DNAuria (BK(−), yellow), and those with BKPyV DNAuria (BK(+), orange). The first two principal components accounted for a total of 87.6% of the variance in the dataset. Specifically, PC1 explained 67.9% of the total variance, and PC2 explained 19.6%.

Finally, based on differently expressed proteins, we performed PCA comparing BK(−), BK(+), and HC (Figure 8). The corresponding Random Forest is provided in the Supplementary Materials Figure S3. The two clusters showed an overlap, yet with a discernible separation between BK(+) and BK(−), and a clear distinction between these two and HC.

**Figure 9:**
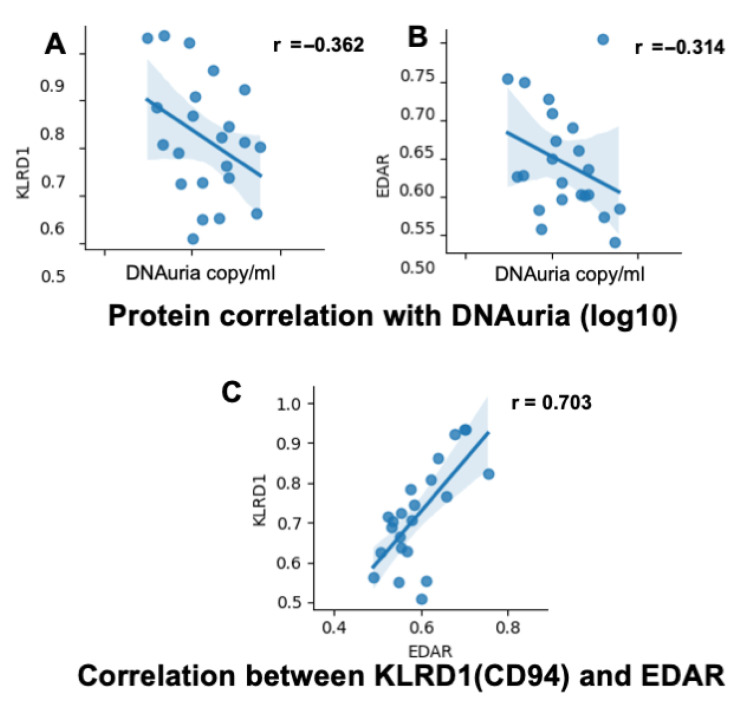
Correlations of urinary immune-related proteins with BK polyomavirus DNAuria and with each other. (**A**) Negative correlation between urinary KLRD1 levels and BK polyomavirus DNAuria (log_10_ copies/mL). (**B**) Negative correlation between urinary EDAR levels and BK polyomavirus DNAuria (log_10_ copies/mL). (**C**) Positive correlation between EDAR and KLRD1 expression levels in urine. Shaded areas represent 95% confidence intervals of the regression lines.

To explore the relationship with BKPyV DNAuria more closely, we examined the correlations among urinary expression levels of EDAR, and KLRD1, since these two proteins achieved over 90% detectability in all samples. Notably, EDAR and KLRD1 show a strong positive correlation with each other and appear to decrease in parallel with the load of BKPyV DNAuria (Figure 9). However, the statistical significance was not indicated.

## 4. Discussion

The study compared the immune response profile present in the urine of several groups: healthy individuals, patients with stage 5 chronic kidney disease, and kidney transplant recipients, including those with BKPyV DNAuria. We hypothesized that urinary replication of BKPyV is associated with this profile in kidney transplant recipients and shifts it away from healthy individuals. We further assumed that these shifts are possibly related to controlling the latent BKPyV infection. The markers included in the applied Olink Target 96 Immune Response panel revealed differentiated detectability in urine; however, a number of these displayed high detectability rates of over 90%, including KLRD1 (CD94) and EDAR. Notably further, CXADR was entirely undetectable in the HC group; however, it was relatively highly expressed in all other groups. Despite the overall higher protein expression in the CKD group, KLRD1 (CD94) appeared to be the only one upregulated in kidney transplant recipients compared both to healthy controls and CKD. The differences in proteomic profiles between kidney transplant recipients and healthy controls were shown to be more evident in BKPyV-DNA-positive than in BKPyV-DNA-negative patients. Despite the fact that no unique profile was identified exclusively for BKPyV DNAuria, a set of four proteins—EDAR, KLRD1 (CD94), PTH1R, and CXADR—appeared to differentiate between BKPyV-DNA-positive and BKPyV-DNA-negative transplant recipients, indicating a measurable shift in the PCA plot. KLRD1 (CD94) and EDAR reveal a statistically insignificant, but evident, negative relationship with DNAuria load, and a positive relationship with each other.

KLRD1 (CD94) is a key receptor in the NK cell repertoire, forming heterodimers with NKG2 family members—either inhibitory (NKG2A) or activating (NKG2C)—to recognize HLA-E, a non-classical MHC class I molecule [11,12]. The CD94/NKG2A complex contributes to NK cell licensing, ensuring self-tolerance and functional competence through interaction with self-MHC, while CD94/NKG2C promotes cytotoxicity [13,14]. CD94 is also expressed on CD8^+^ T cells, where it modulates responses to viral antigens. Gunturi et al. showed that CD94/NKG2 signaling in CD8^+^ T cells regulates both effector function and survival via Qa-1, the murine homolog of HLA-E [15]. In our study, urinary KLRD1 (CD94) expression was significantly elevated in kidney transplant recipients compared to both CKD patients and healthy controls. Still, it was markedly reduced in BKPyV-positive recipients, correlating with viral load and strongly distinguishing BKPyV-infected individuals from healthy controls. Prior studies by the Lukacher group support the role of CD94 in antiviral immunity, particularly in polyomavirus infections. Byers et al. reported CD94/NKG2A expression on CD8^+^ T cells during persistent polyomavirus infection, which is linked to proliferative potential and IL-2 production [16]. Wojtasik et al. demonstrated that CD94/NKG2 expression is induced by TCR signaling, reflecting antigen-specific activation [17]. Further evidence from Fang et al. indicates that CD94 is essential for NK cell–mediated viral defense, as CD94-deficient mice showed increased susceptibility to lethal infection [18]. Nevertheless, the specific CD94/NKG2C’s role in BK polyomavirus remains uncharacterized [19]. Its expression—like that of CD94/NKG2A—is known to be influenced by tacrolimus treatment, aligning with the overall upregulation observed in transplant recipients in our cohort [20].

Among the downregulated proteins, EDAR also showed significantly lower expression in the BKPy-DNA-positive group. It also revealed a strong relationship with KLRD1 (CD94) and with the viral load. The EDAR protein is a transmembrane receptor involved in the signaling pathway associated with ectodermal development. It plays a crucial role in the formation of various structures derived from the ectoderm, such as hair, teeth, sweat glands, and sebaceous glands. Hence, it potentially impacts the body’s protective barriers and its ability to combat infections [21,22]. At the molecular level, EDAR activates the NF-κB pathway, influencing the expression of genes involved in both innate and adaptive immunity, including those related to antiviral immunity [23,24]. Although direct studies on the impact of EDAR protein levels on viral infections are limited, we hypothesize that there are several mechanisms through which EDAR downregulation may indirectly influence susceptibility to viral infections, by influencing innate rather than adaptive immunity. Notably, EDAR expression has also been reported in The Human Protein Atlas in renal tubular epithelial cells, although this aspect remains poorly characterized in the literature. Given EDAR’s emerging role in immune signaling, this finding may indicate a compromised epithelial immune function in the renal tubular microenvironment, potentially facilitating viral persistence or impaired antiviral responses in the urinary tract. Interestingly, EDAR detection rates exceeded 95% in all samples.

CXADR is widely expressed in different locations, including epithelia, muscles, brain, heart, lungs, and kidneys [25]. It was primarily recognized as a transmembrane receptor protein that enables cell invasion with coxsackieviruses and adenoviruses through the interactions with the respective viral capsid proteins [26,27]. However, CXADR exhibits diverse functions in cellular physiology, such as regulating the cell’s adhesion, migration, differentiation, and signal transduction [28]. It has also been described as involved in intercellular junction [29]. To date, CXADR has not been described in the context of infections with polyomavirus, which itself is known to replicate in the epithelium of renal tubules [30]. High detectability rate of CXADR in both post-transplant groups and CKD, in contrast to the healthy controls, may indicate increased epithelial cell turnover associated with chronic kidney disease and the kidney transplantation group. The upregulation observed during BKPyV DNAuria may be triggered by the infection itself, and this aligns with the fact that BK virus replicates in epithelial cells. We find it remarkable that it plays a role in regulating innate immune responses at the epithelial level by stimulating gamma delta lymphocytes to produce cytokines, which, in turn, influence epithelial regeneration processes [31,32]. Moreover, in the epithelial setting specifically, CXADR deficiency has also been found to be associated with reduced inflammatory responses, including fewer inflammatory cell infiltrations and decreased cytokine release [33]. Considering all the above, we are prone to hypothesize that the role of CXADR in BKPyV infection is to contribute to immunity itself or cellular turnover, rather than acting as a virus receptor molecule. However, the implications of this finding for the graft and the course of BKPyV infection in kidney transplant recipients remain unclear. Further studies are necessary to explore its role in the progression of BKPyV infection and its potential clinical utility, especially given the growing interest in this protein in various contexts. These include recent and impactful data on the role of oncogenic *KRAS* mutations, which induce CXADR expression, facilitate enterovirus infections, and accelerate the progression of pancreatic cancer [34]. Similarly, CXADR has been implicated in type 1 diabetes, where the presence of enterovirus was detected in β cells of pancreatic islets [35].

PTH1R is a receptor for parathyroid hormone (PTH), a peptide involved in regulating calcium-phosphate metabolism. This receptor is primarily present in osteoblastic cells, where it acts in conjunction with parathyroid hormone to stimulate bone resorption and increase the blood calcium level [36]. The protein PTH1R reveals certain connections with the immune system through its interaction with parathyroid hormone. Although it is not directly involved in the immune response in the same manner as typical immune system receptors, studies suggest that PTH and its receptor may influence immunity. These indicate that PTH affects the activity of T lymphocytes and dendritic cells, which mainly refers to cytokine secretion. However, the full extent of the interaction between PTH and PTH1R with the immune system, as well as the mechanisms of these interactions, requires further research [37,38]. The influence of chronic kidney disease on PTH metabolism and its impact on the PTH1R level cannot be omitted [39]. Some of the available data demonstrate increased expression of PTH1R in CKD [40], but other evidence appears contradictory [41]. In our study, as shown in Supplementary Materials Table S1, PTH1R had the highest detectability in the CKD group, supporting the hypothesis that its upregulation is more likely related to disturbed calcium-phosphate metabolism rather than immune activity. Therefore, the observed expression pattern in CKD is biologically plausible.

The study has several limitations, primarily due to its single-center character. Therefore, selection bias cannot be excluded, as the study population was derived from available urine samples meeting predefined eligibility criteria. Measurement bias may arise from technical limitations inherent to targeted proteomic platforms and includes variability in protein detectability and reliance on relative NPX values. Nevertheless, the NPX values are generated through a rigorous, multi-step internal normalization process and represent relative protein abundance. Therefore, these are commonly used as an input for profiling and exploratory strategies as implemented in this study. The variety of detection rates for different proteins, is typically observed in urine, which makes urinary investigations somewhat challenging. However, in this study, standardized sample processing and quality control procedures were applied to minimize analytical variability. Since the normalized expression values represent the excellent potential for profiling algorithms, we included detectability as an additional variable. In our study, the large proportion of proteins surpassed the 50% detectability threshold, though often only within individual groups. So, we established the threshold of 50% detectability in at least one group. Still, from the point of view of antiviral immunity, the most interesting proteins, such as KLRD1 (CD94) and EDAR, showed an outstanding detectability rate of over 90% in all samples. These also showed a relationship with the viral load; however, possibly due to the sample size, we could not establish statistical significance of this finding. The observational design of the study precludes causal inference and limits conclusions about associations found at a single time point. Nevertheless, this observation suggests that the shifts we identified, particularly in KLRD1 (CD94), reflect a deeper level of immunity against BKPyV under immunosuppression.

As highlighted in “The Second International Consensus Guidelines on the Management of BK Polyomavirus in Kidney Transplantation” [2], occasional detection of BKPyV DNA in urine is possible both in healthy individuals and kidney transplant recipients. This concern has been addressed in both Random Forrest and PCA, which emphasized that the BK(−) and BK(+) groups diverge from healthy controls in distinct ways. So, it was in the PCA plot, which involved the four proteins of significantly different expression between BK(+), BK(−), and HC, and further by indicating the relationship between KLRD1 (CD94), EDAR, and DNAuria load. Indeed, the clinical consensus also highlights the significance of BKPyV DNAemia in the management of BKPyV reactivation. However, investigating the progression from DNAuria to DNAemia remained beyond the aim of this study because it primarily represents an explorative character and focuses on the local consequences of urinary BKPyV replication.

## 5. Conclusions

In conclusion, our study highlights the proteomic diversity among the examined groups. Within the immune response panel used, most of the detected proteins were upregulated in the CKD group. Not surprisingly, kidney transplantation appeared to attenuate this effect, making the urinary proteomic profile of kidney transplant recipients closer to that of healthy controls. In contrast, the presence of BKPyV DNAuria shifted these profiles away from healthy controls. All the above indicate that viral replication introduces a specific biological imprint that overlays the transplant-related immune modulation. Although the number of significantly dysregulated proteins between the BK(+) and BK(−) groups is limited, the ones we identified, especially KLRD1 (CD94), carry a high biological relevance reflected in the previous basic research on BKPyV latency. Hence, we propose CD94 as a candidate for further investigation on the dynamics of BKPyV reactivation in the kidney transplant recipients.

## Figures and Tables

**Table 1:** Group characteristics.

	TX	CKD	HC
Samples (number)	104	18	15
Patients (number)	51	18	15
Female (samples)	43	12	8
Male (samples)	61	6	7
Female (%)	41.4	66.7	53.3
Male (%)	58.6	33.3	46.7
Age (mean)	48.9 ± 13.4	43 ± 10.4	24 ± 3.5

TX: kidney transplant recipients; CKD: patients with chronic kidney disease; HC: healthy controls. Age is presented as mean ± standard deviation. Percentages refer to the distribution of samples by sex.

**Table 2:** Glomerular filtration rate values.

	BK(−)	BK(+)
Samples (number)	75	29
Mean GFR (mL/min/1.73 m^2^) ± SD	62.3 ± 20.7	61.5 ± 19.9
Median	61.6	60.5
Range (min-max)	15–100.6	20.4–90.9
*p*-value	9.94	

BK(−): kidney transplant recipients without BKPyV; BK(+): kidney transplant recipients with BKPyV DNAuria. GFR: glomerular filtration rate; values are presented as mean ± standard deviation, median, and range. Normality was assessed using the Shapiro–Wilk test. The *p*-value was calculated using the unpaired Student’s *t*-test.

**Table 3:** KEGG pathway associations for proteins EDAR, PTH1R, CXADR, and KLRD1. KEGG pathway analysis was performed for four selected proteins: EDAR, PTH1R, CXADR, and KLRD1. Data were retrieved using KEGG Mapper and literature-based validation.

EDAR	PTH1R	CXADR	KLRD1
Cytokine-cytokine receptor interaction	Neuroactive ligand-receptor interaction	Viral myocarditis	Antigen processing and presentation
NF-kappa B signaling pathway	Parathyroid hormone synthesis, secretion, and action	Virion-Adenovirus	Natural killer cell-mediated cytotoxicity
	Endocrine and other factor-regulated calcium reabsorption		Graft-versus-host disease

## Data Availability

All relevant data are within the manuscript and its Supplement Materials. Additional data supporting the findings of this study are available from the corresponding author upon reasonable request.

## Supplementary Materials

The following supporting information can be downloaded at: https://www.mdpi.com/article/10.3390/medicina62020240/s1, Figure S1: Random Forest BK vs. HC. The bar plot displays the relative importance of individual urinary immune proteins in distinguishing between study groups, as determined by the Random Forest algorithm. Higher values indicate a greater contribution of a protein to the classification model. KLRD1, HEXIM1, and CXADR emerged as the top-ranked proteins; Figure S2: Random Forest TX vs. HC. The bar plot displays the relative importance of individual urinary immune proteins in distinguishing between study groups, as determined by the Random Forest algorithm. Higher values indicate a greater contribution of a protein to the classification model. BTN3A2, LILRB4, and CLEC4A emerged as the top-ranked proteins; Figure S3: Random Forest for BK vs. TX vs. HC- differently expressed. The bar plot displays the relative contribution of four proteins (CXADR, PTH1R, KLRD1, EDAR) to group classification as determined by the Random Forest algorithm; Table S1: List of protein with detection percentage; Table S2: Statistical analysis by regression modeling—HC vs. TX; Table S3: Statistical analysis by regression modeling—CKD vs. TX; Table S4: Statistical analysis by regression modeling—CKD vs. HC.

## Abbreviations

The following abbreviations are used in this manuscript:

BKPyV	BK virus
BKPyV-DNA	BK Polyomavirus DNA
TX	Kidney transplant recipients
CKD	Chronic kidney disease
HC	Healthy controls
PEA	Proximity extension assay
KLRD1	Killer cell lectin-like receptor subfamily D member 1
BKVAN	BK virus-associated nephropathy
PCR	polymerase chain reaction
NPX	Normalized Protein Expression
DEPs	Differentially expressed proteins
RF	Random Forest
PCA	Principal Component Analysis
LOD	Limit of detection
GLM	General linear model
BK(+)	Patients with BKPyV DNAuria
BK(−)	Patients without BKPyV DNAuria
GFR	Glomerular filtration rate
Z-scores	Standardized expression levels
CLEC7A	C-type lectin domain family 7 member A
CD83	CD83 antigen
ITGB6	Integrin beta 6
PRDX1	Peroxiredoxin 1
HNMT	Histamine N-methyltransferase
CDSN	Corneodesmosin
EDAR	Tumor necrosis factor receptor superfamily member EDAR (EDAR)
BTN3A2	Butyrophilin subfamily 3 member A2
STC1	Stanniocalcin-1
FAM3B	Protein FAM3B
KRT19	Keratin, type 1cytoskeletal 19
CLEC4A	C-type lectin domain family 4 member A
AREG	Amphiregulin
PTH1R	Parathyroid hormone receptor 1
CXADR	Coxsackievirus and adenovirus receptor
LILRB4	Leukocyte immunoglobulin like receptor B4
DFFA	DNA fragmentation factor subunit alpha
DCBLD2	Discoidin, CUB and LCCL domain-containing protein
PADI2	Peptidyl arginine deiminase 2
CCL11	C-C motif chemokine 11
NKG2A	Killer cell lectin-like receptor subfamily C1
NKG2C	Killer cell lectin-like receptor subfamily C2/3
CD94	CD94 antigen
HLA-E	Major histocompatibility complex, class I, E
MHC	Major histocompatibility complex
NK	Natural killer cell
TCR	T-cell antigen receptor alpha
PTH	Parathormone

## Appendix A

**Table A1:** Parameters of DNAuria.

	DNAuria (Copy/mL)
Min. value	290
Max. value	772,500,000
Average	64,406,589.6
Mean	1,450,000
SD	174,876,401.5

## Appendix C

**Table A2:** List of proteins shared between groups as shown in the Venn diagram.

Proteins Shared Exclusively Among the CKD, BK(+), and BK(−) Groups	Common Proteins (Shared Across All Groups)
dual adapter for phosphotyrosine and 3-phosphotyrosine and 3-phosphoinositide (DAPP1)	leukocyte immunoglobulin-like receptor subfamily B member 4 (LILRB4)
ATP-dependent RNA helicase DDX58 (DDX58)	butyrophilin subfamily 3 member A2 (BTN3A)
interleukin 12 receptor beta-1 (IL12RB1)	keratin, type 1cytoskeletal 19 (KRT19)
tryptase alpha/beta 1 (TPSAB1)	C-type lectin domain family 7 member A (CLEC7A)
Fanconi anaemia group J protein (BACH1)	histamine N-methyltransferase (HNMT)
fibroblast growth factor 2 (FGF2)	lysosomal-associated membrane protein 3 (LAMP3)
mannan-binding lectin serine protease 1 (MASP1)	corneodesmosin (CDSN)
eukaryotic translation initiation factor 4 gamma 1 (EIF4G1)	peroxiredoxin-5, mitochondrial (PRDX5)
interferon regulatory factor 9 (IRF9)	eotaxin (CCL11)
hematopoietic cell-specific Lyn substrate 1 (HCLS1)	killer cell lectin-like receptor subfamily D member 1 (KLRD1)
zinc finger and BTB domain-containing protein 16 (ZBTB16)	interleukin 6 (IL6)
PC4 and SFRS1-interacting protein (PSIP1)	stanniocalcin-1 (STC1)
C-type lectin domain family 4 member C (CLEC4C)	DNA fragmentation factor subunit alpha (DFFA)
mast cell immunoglobulin-like receptor 1 (MILR)	C-type lectin domain family 4 member A (CLEC4A)
natural cytotoxicity triggering receptor 1 (NCR1)	protein-arginine-deiminase type 2 (PADI2)
C-type lectin domain family 4 member D (CLEC4D)	amphiregulin (AREG)
coxsackievirus and adenovirus receptor (CXADR)	tumor necrosis factor receptor superfamily member EDAR (EDAR)
	discoidin, CUB and LCCL domain containing 2 (DCBLD2)
	beta-galactosidase (GLB1)
	protein FAM3B (FAM3B)
	CD83 antigen (CD83)
	parathyroid hormone receptor 1 (PTH1R)
	integrin beta 6 (ITGB6)
	protein HEXIM1 (HEXIM1)
	peroxiredoxin 1 (PRDX1).

## References

[1] Hirsh H.H., Steiger J. (2003). Polyomavirus BK. Lancet Infect. Dis..

[2] Kotton C.N., Kamar N., Wojciechowski D., Eder M., Hopfer H., Randhawa P., Sester M., Comoli P., Silva H.T., Knoll G. (2024). The second international consensus guidelines on the management of BK Polyomavirus in kidney transplantation. Transplantation.

[3] Gardner S.D., Field A.M., Coleman D.V., Hulme B. (1971). New human papovavirus (B.K.) isolated from urine after renal transplantation. Lancet.

[4] Jamboti J.S. (2006). BK virus nephropathy in renal transplant recipients. Nephrology.

[5] Nickeleit V., Singh H.K. (2015). Polyomaviruses and disease: Is there more to know than viremia and viruria?. Curr. Opin. Organ Transplant..

[6] Lauver M.D., Katz Z.E., Markus H., Derosia N.M., Jin G., Ayers K.N., Butic A.B., Bushey K., Abendroth C.S., Liu D.J. (2025). The CXCR6–CXCL16 axis mediates T cell control of polyomavirus infection in the kidney. PLoS Pathog..

[7] Sandybayev N., Belloussov V., Strochkov V., Solomadin M., Granica J., Yegorov S. (2022). Next Generation Sequencing Approaches to Characterize the Respiratory Tract Virome. Microorganisms.

[8] Qian L., Sun R., Aebersold R., Bühlmann P., Sander C., Guo T. (2024). AI-empowered perturbation proteomics for complex biological systems. Cell Genom..

[9] Breiman L. (2001). Random forests. Mach. Learn..

[10] Urbiola-Salvador V., Jabłońska A., Miroszewska D., Huang Q., Duzowska K., Drężek-Chyła K., Zdrenka M., Śrutek E., Szylberg Ł., Jankowski M. (2023). Plasma protein changes reflect colorectal cancer development and associated inflammation. Front. Oncol..

[11] Chang C., Rodriguez A., Carretero M., López-Botet M., Phillips J.H., Lanier L.L. (1995). Molecular characterization of human CD94: A type II membrane glycoprotein related to the C-type lectin superfamily. Eur. J. Immunol..

[12] Braud V., Allan D., O’Callaghan C., Soderstrom K., D’Andrea A., Ogg G.S., Lazetic S., Young N.T., Bell J.I., Phillips J.H. (1998). HLA-E binds to natural killer cell receptors CD94/NKG2A, B and C. Nature.

[13] Yokoyama W.M., Kim S. (2006). Licensing of natural killer cells by self-major histocompatibility complex class I. Immunol. Rev..

[14] Kim S., Poursine-Laurent J., Truscott S.M., Lybarger L., Yun-Jeong S., Yang L., French A.R., Sunwoo J.B., Lemieux S., Hansen T.H. (2005). Licensing of natural killer cells by host major histocompatibility complex class I molecules. Nature.

[15] Gunturi A., Berg R.E., Forman J. (2004). The role of CD94/NKG2 in innate and adaptive immunity. Immunol. Res..

[16] Byers A.M., Andrews N.P., Lukacher A.E. (2006). CD94/NKG2A expression is associated with proliferative potential of CD8 T cells during persistent Polyoma virus infection. J. Immunol..

[17] Wojtasik M., Jones C.M., Sullivan L.C., Winterhalter A.C., Carbone F.R., Brooks A.G. (2004). Persistent expression of CD94/NKG2 receptors by virus-specific CD8 T cells is initiated by TCR-mediated signals. Int. Immunol..

[18] Fang M., Orr M.T., Spee P., Egebjerg T., Lanier L.L., Sigal L.J. (2011). CD94 is essential for NK cell-mediated resistance to a lethal viral disease. Immunity.

[19] Aubry A., Demey B., Castelain S., Helle F., Brochot E. (2024). The value and complexity of studying cellular immunity against BK Polyomavirus in kidney transplant recipients. J. Clin. Virol..

[20] Tanaka J., Toubai T., Iwao N., Tsutsumi Y., Kato N., Miura Y., Shigematsu A., Hirate D., Ota S., Asaka M. (2005). The immunosuppressive agent FK506 enhances the cytolytic activity of inhibitory natural killer cell receptor (CD94/NKG2A)-expressing CD8 T cells. Transplantation.

[21] Koppinen P., Pispa J., Laurikkala J., Thesleff I., Mikkola M.L. (2001). Signaling and subcellular localization of the TNF receptor Edar. Exp. Cell Res..

[22] Kowalczyk-Quintas C., Schneider P. (2014). Ectodysplasin A (EDA)—EDA receptors signaling and its pharmacological modulation. Cytokine Growth Factor Rev..

[23] Hoffmann A., Baltimore D. (2006). Circuitry of nuclear factor κB signaling. Immunol. Rev..

[24] Liu T., Zhang L., Joo D., Sun S.C. (2017). NF-κB signaling in inflammation. Signal Transduct. Target. Ther..

[25] Wehbi A., Kremer E.J., Dopeso-Reyes I.G. (2020). Location of the cell adhesion molecule “Coxsackievirus and Adenovirus Receptor” in the adult mouse brain. Front. Neuroanat..

[26] Coyne C.B., Bergelson J.M. (2006). Virus-induced Abl and Fyn kinase signals permit coxsackievirus entry through epithelial tight junctions. Cell.

[27] Bergelson J.M., Cunningham J.A., Droguett G., Kurt-Jones E.A., Krithivas A., Horwitz M.S., Crowell R.L., Finberg R.W. (1997). Isolation of a common receptor for coxsackie B viruses and adenoviruses 2 and 5. Science.

[28] Houri N., Huang K.C., Nalbantoglu J. (2013). The coxsackievirus and adenovirus receptor (CAR) undergoes ectodomain shedding and regulated intramembrane proteolysis (RIP). PLoS ONE.

[29] Raschperger E., Neve E.P., Wernerson A., Hultenby K., Pettersson R.F., Majumdar A. (2008). The coxsackie and adenovirus receptor (CAR) is required for renal epithelial differentiation within the zebrafish pronephros. Dev. Biol..

[30] Moriyama T., Sorokin A. (2008). Intracellular trafficking pathway of BK virus in human renal proximal tubular epithelial cells. Virology.

[31] Cohen C.J., Shieh J.T.C., Pickles R.J., Okegawa T., Hsieh J.T., Bergelson J.M. (2001). The coxsackievirus and adenovirus receptor is a transmembrane component of the tight junction. Proc. Natl. Acad. Sci. USA.

[32] Tomko R.P., Xu R., Philipson L. (1997). HCAR and MCAR: The human and mouse cellular receptors for subgroup C adenoviruses and group B coxsackieviruses. Proc. Natl. Acad. Sci. USA.

[33] Ortiz-Zapater E., Bagley D.C., Hernandez V.L., Roberts L.B., Maguire T.J.A., Voss F., Mertins P., Kirchner M., Peset-Martin I., Woszczek G. (2022). Epithelial coxsackievirus adenovirus receptor promotes house dust mite-induced lung inflammation. Nat. Commun..

[34] Bastea L.I., Liu X., Fleming A.K., Pandey V., Döppler H., Edenfield B.H., Krishna M., Zhang L., Thompson E.A., Grandgenett P.M. (2024). Coxsackievirus and adenovirus receptor expression facilitates enteroviral infections to drive the development of pancreatic cancer. Nat. Commun..

[35] Vehik K., Lynch K.F., Wong M.C., Tian X., Ross M.C., Gibbs R.A., Ajami N.J., Petrosino J., Rewers M., Toppari J. (2019). Prospective virome analyses in young children increased genetic risk for type 1 diabetes. Nat. Med..

[36] Abou-Samra A.B., Jüppner H., Kong X.F., Schipani E., Iida-Klein A., Karga H., Urena P., Gardella T.F., Potts J.T., Kronenberg H.M. (1994). Structure, function, and expression of the receptor for parathyroid hormone and parathyroid hormone-related peptide. Adv. Nephrol. Necker Hosp..

[37] Weitzmann M.N., Pacifici R. (2005). The role of T lymphocytes in bone metabolism. Immunol. Rev..

[38] D’Amelio P., Grimaldi A., Di Bella S., Brianza S.Z.M., Cristofaro M.A., Tamone C., Giribaldi G., Ulliers D., Pescarmona G.P., Isaia G. (2008). Estrogen deficiency increases osteoclastogenesis up-regulating T cells activity: A key mechanism in osteoporosis. Bone.

[39] Evenepoel P., Bover J., Torres P.U. (2016). Parathyroid hormone metabolism and signaling in health and chronic kidney disease. Kidney Int..

[40] Pereira R.C., Delany A.M., Khouzam N.M., Bowen R., Freymiller E., Salusky I.B., Wesseling-Perry K. (2015). Primary osteoblast-like cells from patients with end-stage kidney disease reflect gene expression, proliferation, and mineralization characteristics ex vivo. Kidney Int..

[41] Picton M.L., Moore P.R., Mawer E.B., Houghton D., Freemont A.J., Hutchinson A.J., Gokal R., Hoyland J.A. (2000). Down-regulation of human osteoblast PTH/PTHrP receptor mRNA in end-stage renal failure. Kidney Int..

