# Supplementary material for: Explorative Insights into Local Immune Response to BK Virus—A Cross-Sectional Study in Urine Samples Between Transplant Recipients and Non-Immunocompromised Hosts
Source: Medicina (Kaunas). 2026 Jan 23;62(2):240. doi: 10.3390/medicina62020240 (PMC12941888; doi:10.3390/medicina62020240)

SUPPLEMENTARY MATERIALS

Table S1. List of protein with detection percentage

Protein	BK	TX	CKD	HC
PRDX1	100	100	100	100
KLRD1	100	100	94,4	100
CLEC7A	100	94,8	94,4	92,9
CD83	100	100	100	100
ITGB6	100	100	100	100
EDAR	96,6	96,6	100	100
CDSN	96,6	98,3	100	100
HNMT	96,6	94,8	100	100
BTN3A2	89,7	89,7	94,4	85,7
LAMP3	86,2	96,6	100	100
STC1	75,9	75,9	100	100
FAM3B	72,4	81	100	85,7
LILRB4	69	70,7	88,9	21,4
CCL11	69	74,1	72,2	35,7
KRT19	65,5	60,3	94,4	28,6
CLEC4A	55,2	69	94,4	28,6
CXADR	55,2	62,1	88,9	0
GLB1	48,3	41,4	77,8	14,3
HEXIM1	44,8	37,9	88,9	7,1
AREG	44,8	72,4	94,4	28,6
IL6	37,9	27,6	77,8	7,1
PTH1R	31	24,1	94,4	14,3
CLEC4D	31	12,1	88,9	0
DCBLD2	31	31	66,7	64,3
PRDX5	27,6	17,2	77,8	7,1
JUN	24,1	8,6	77,8	7,1
DFFA	24,1	20,7	77,8	28,6
NCR1	20,7	25,9	77,8	0
MILR1	17,2	5,2	55,6	0
CNTNAP2	13,8	15,5	61,1	14,3
FGF2	13,8	1,7	38,9	0
CKAP4	13,8	1,7	83,3	14,3
MGMT	13,8	0	55,6	0
LAG3	13,8	1,7	38,9	7,1
ZBTB16	10,3	1,7	16,7	0
DGKZ	10,3	1,7	0	0
DCTN1	10,3	10,3	50	7,1
PADI2	10,3	5,2	22,2	7,1
TPSAB1	6,9	3,4	77,8	0

HCLS1	6,9	1,7	38,9	0
PRDX3	6,9	1,7	16,7	7,1
CLEC6A	6,9	0	38,9	0
DDX58	6,9	1,7	22,2	0
PSIP1	3,4	1,7	5,6	0
IRF9	3,4	3,4	5,6	0
CLEC4C	3,4	3,4	33,3	0
TRIM5	3,4	0	33,3	0
GALNT3	3,4	0	5,6	0
TRAF2	3,4	0	5,6	0
TRIM21	3,4	0	22,2	0
NTF4	3,4	0	22,2	0
EGLN1	3,4	0	5,6	0
NFATC3	3,4	0	5,6	0
EIF5A	3,4	1,7	0	0
EIF4G1	3,4	1,7	66,7	0
SRPK2	3,4	0	44,4	14,3
BACH1	3,4	1,7	44,4	0
PIK3AP1	3,4	0	27,8	0
SPRY2	3,4	5,2	11,1	7,1
FCRL6	3,4	0	33,3	0
DAPP1	3,4	1,7	5,6	0
MASP1	3,4	1,7	33,3	0
IL12RB1	3,4	3,4	44,4	0
TANK	3,4	1,7	11,1	14,3
PPP1R9B	0	0	33,3	0
IRAK4	0	1,7	5,6	0
CLEC4G	0	0	38,9	0
IRAK1	0	0	50	0
DPP10	0	1,7	5,6	7,1
ITGA6	0	1,7	22,2	0
FXYD5	0	5,2	44,4	0
ITM2A	0	0	55,6	0
LY75	0	0	0	0
CD28	0	1,7	0	0
BIRC2	0	0	16,7	0
HSD11B1	0	0	0	0
NF2	0	0	5,6	0
PLXNA4	0	0	0	0
SH2B3	0	0	0	0
FCRL3	0	0	0	0
PRKCQ	0	1,7	5,6	0
TREM1	0	0	16,7	0
IL10	0	0	5,6	0
ARNT	0	0	0	0

SH2D1A	0	0	27,8	0
ICA1	0	0	5,6	0
CXCL12	0	0	0	0
IFNLR1	0	0	0	0
SIT1	0	0	5,6	14,3
ITGA11	0	0	0	7,1
KPNA1	0	0	0	0
IL5	0	0	0	0

Figure S1. Random Forest BK vs HC. The bar plot displays the relative importance of individual urinary immune proteins in distinguishing between study groups, as determined by the Random Forest algorithm. Higher values indicate a greater contribution of a protein to the classification model. KLRD1, HEXIM1, and CXADR emerged as the top-ranked proteins.

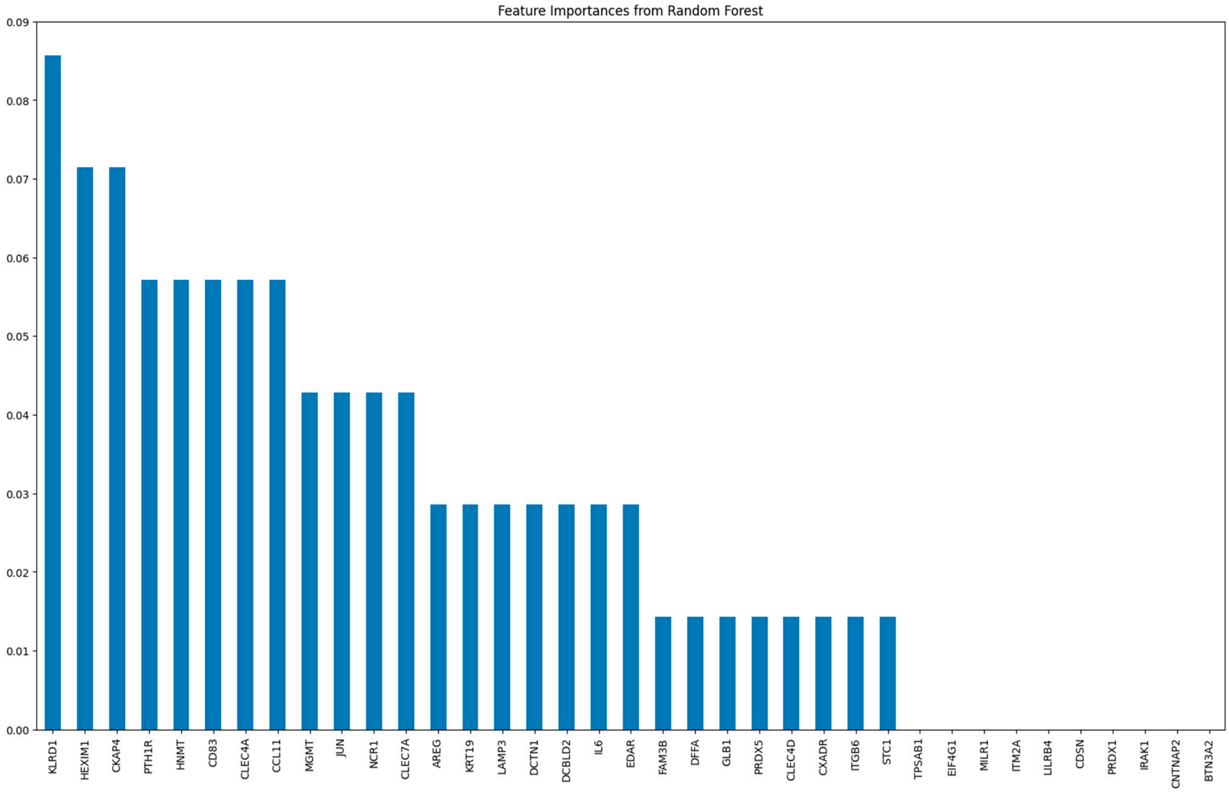

Figure S2. Random Forest TX vs HC. The bar plot displays the relative importance of individual urinary immune proteins in distinguishing between study groups, as determined by the Random Forest algorithm. Higher values indicate a greater contribution of a protein to the classification model. BTN3A2, LILRB4, and CLEC4A emerged as the top-ranked proteins.

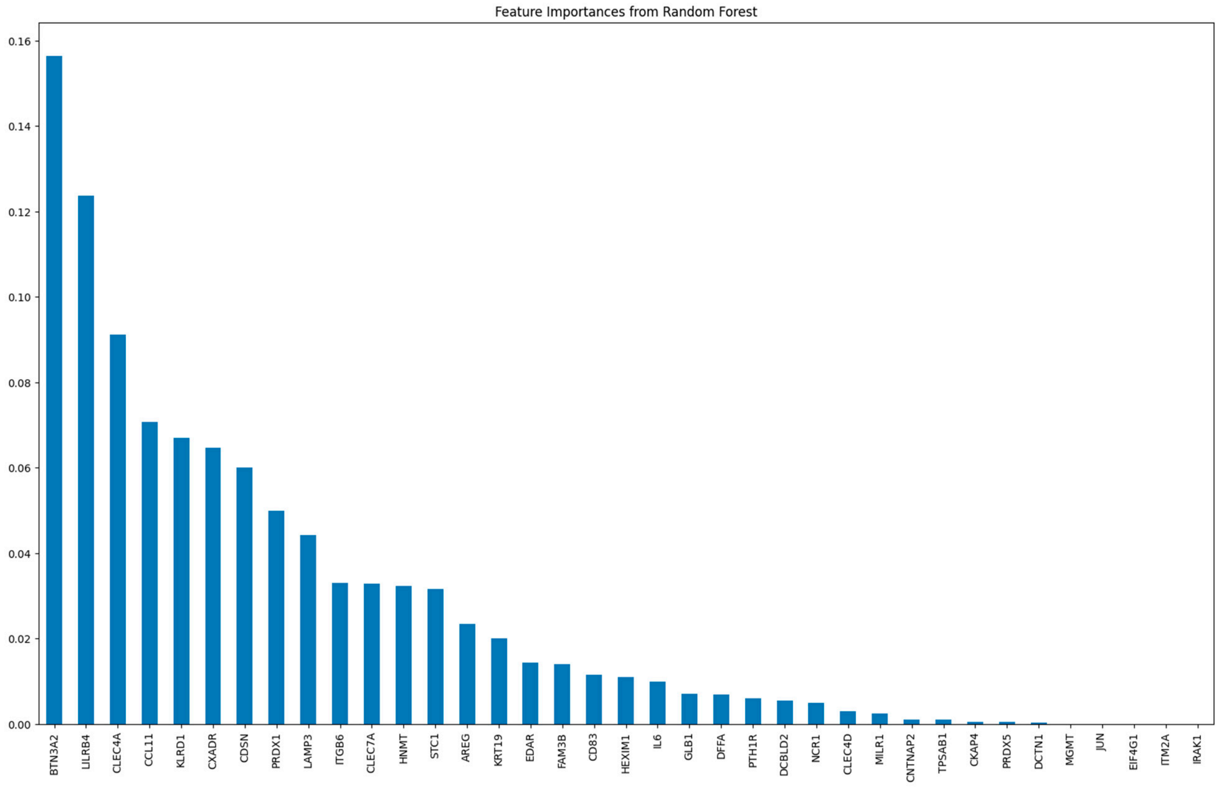

Table S2. Statistical analysis by regression modeling – HC vs TX

prot	mean.ref	mean.oi	se.ref	se.oi	estimate	SE	p.value	diff
KLRD1	3.508	5.074	NA	NA	1.567	0.502	0.007	1.567
BTN3A2	2.006	3.019	NA	NA	1.013	0.501	0.045	1.013
ITGB6	4.495	3.850	NA	NA	-0.645	0.287	0.078	-0.645
LILRB4	2.200	3.881	NA	NA	1.681	1.034	0.108	1.681
CD83	3.595	3.091	NA	NA	-0.504	0.281	0.112	-0.504
EDAR	3.702	3.280	NA	NA	-0.422	0.239	0.121	-0.422
CLEC7A	5.771	5.249	NA	NA	-0.522	0.323	0.164	-0.522
CDSN	4.563	5.007	NA	NA	0.444	0.364	0.224	0.444
PRDX1	3.262	4.166	NA	NA	0.903	0.631	0.249	0.903
CCL11	2.197	2.696	NA	NA	0.499	0.476	0.297	0.499

Table S3. Statistical analysis by regression modeling – CKD vs TX

prot	mean.ref	mean.oi	se.ref	se.oi	estimate	SE	p.value	diff
CLEC4A	3.444	2.115	NA	NA	-1.329	0.189	0.000	-1.329
CDSN	6.470	5.007	NA	NA	-1.463	0.281	0.000	-1.463
KRT19	5.525	2.686	NA	NA	-2.839	0.508	0.000	-2.839
HNMT	7.697	4.641	NA	NA	-3.056	0.454	0.000	-3.056
PTH1R	4.018	2.390	NA	NA	-1.628	0.315	0.000	-1.628
STC1	4.080	1.981	NA	NA	-2.099	0.236	0.000	-2.099
FAM3B	5.172	3.205	NA	NA	-1.967	0.327	0.000	-1.967
DCBLD2	3.284	1.401	NA	NA	-1.883	0.281	0.000	-1.883
AREG	4.241	2.084	NA	NA	-2.157	0.397	0.000	-2.157
BTN3A2	5.205	3.019	NA	NA	-2.186	0.377	0.000	-2.186
CD83	3.933	3.091	NA	NA	-0.842	0.217	0.001	-0.842
HEXIM1	2.984	1.621	NA	NA	-1.363	0.376	0.002	-1.363
DFFA	3.377	1.750	NA	NA	-1.627	0.447	0.003	-1.627
EDAR	3.847	3.280	NA	NA	-0.567	0.185	0.008	-0.567
LILRB4	4.965	3.881	NA	NA	-1.084	0.461	0.032	-1.084
IL6	5.124	3.591	NA	NA	-1.533	0.597	0.041	-1.533
CKAP4	5.312	3.703	NA	NA	-1.609	0.776	0.158	-1.609
CLEC7A	5.676	5.249	NA	NA	-0.427	0.249	0.164	-0.427
ITGB6	4.201	3.850	NA	NA	-0.351	0.222	0.173	-0.351
CNTNAP2	2.064	1.531	NA	NA	-0.533	0.343	0.202	-0.533

Table S4. Statistical analysis by regression modeling – CKD vs HC

prot	mean.ref	mean.oi	se.ref	se.oi	estimate	SE	p.value
DCBLD2	1.600	3.284	NA	NA	1.684	0.380	0.000
CLEC4A	1.729	3.444	NA	NA	1.715	0.392	0.000
CDSN	4.563	6.470	NA	NA	1.907	0.413	0.000
STC1	1.924	4.080	NA	NA	2.156	0.340	0.000
HNMT	4.844	7.697	NA	NA	2.853	0.668	0.000
BTN3A2	2.006	5.205	NA	NA	3.199	0.568	0.000
FAM3B	3.481	5.172	NA	NA	1.691	0.493	0.001
AREG	1.477	4.241	NA	NA	2.763	0.830	0.002
KLRD1	3.508	5.153	NA	NA	1.645	0.578	0.008
KRT19	2.552	5.525	NA	NA	2.973	1.049	0.009
PTH1R	1.837	4.018	NA	NA	2.181	0.765	0.010
LILRB4	2.200	4.965	NA	NA	2.765	1.073	0.032
DFFA	2.066	3.377	NA	NA	1.312	0.778	0.150
CNTNAP2	0.787	2.064	NA	NA	1.277	0.722	0.202
CKAP4	3.539	5.312	NA	NA	1.773	1.137	0.205

Figure S3. Random Forest for BK vs TX vs HC- differently expressed. The bar plot displays the relative contribution of four proteins (CXADR, PTH1R, KLRD1, EDAR) to group classification as determined by the Random Forest algorithm.

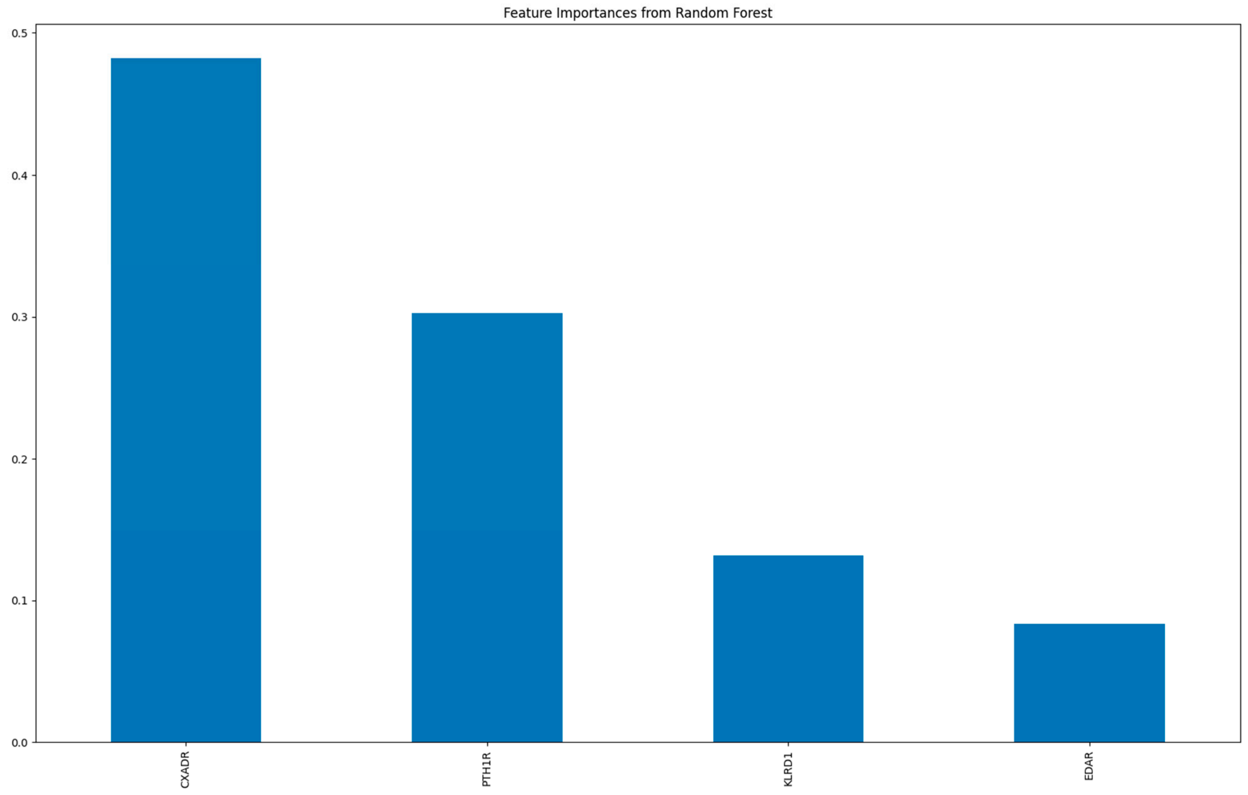

Supplement: Supplementary file 1 [file medicina-62-00240-s001.zip › medicina-4072506-supplementary.pdf]
